# Aqua­bis(4-nitro­benzoato)-κ^2^ *O*,*O*′;κ*O*-(piperidinium-4-carboxyl­ato-κ^2^ *O*,*O*′)cadmium(II)

**DOI:** 10.1107/S160053680901811X

**Published:** 2009-05-20

**Authors:** Run-Wen Zhang, Li-Li Wang, Xiao-Jun Zhao

**Affiliations:** a College of Chemistry and Life Science, Tianjin Key Laboratory of Structure and Performance for Functional Molecule, Tianjin Normal University, Tianjin 300387, People’s Republic of China

## Abstract

In the mixed ligand title compound, [Cd(C_6_H_11_NO_2_)(C_7_H_4_NO_4_)_2_(H_2_O)], which exhibits a discrete mononuclear structure, the Cd^II ^atom is in a distorted octa­hedral geometry, surrounded by five carboxyl­ate O atoms and one coordinated water mol­ecule. The piperdinium ring adopts a chair conformation and the two 4-nitro­benzoate rings are oriented at a dihedral angle of 75.8 (1)°. Inter­molecular O—H⋯O and N—H⋯O hydrogen bonds link the mononuclear entities into a three-dimensional supra­molecular network.

## Related literature

For the framework topologies and potential applications of coordination complexes with mixed ligands, see: Muthu *et al.* (2002 ▶); Fujita *et al.* (1994 ▶); Zheng *et al.* (2004 ▶); Rosi *et al.* (2003 ▶). For 4-piperidine­carboxylic acid as a zwitterion in aqueous solution, see: Mora *et al.* (2002 ▶); and for its ability to act selectively as a bridging or terminal ligand, see: Inomata *et al.* (2002 ▶). For related structures, see: Adams *et al.* (2006*a* ▶,*b* ▶); Podesta & Orpen (2002 ▶); Delgado *et al.* (2001 ▶). For Cd—O bond lengths, see: Inomata *et al.* (2004 ▶); Wang *et al.* (2008 ▶).
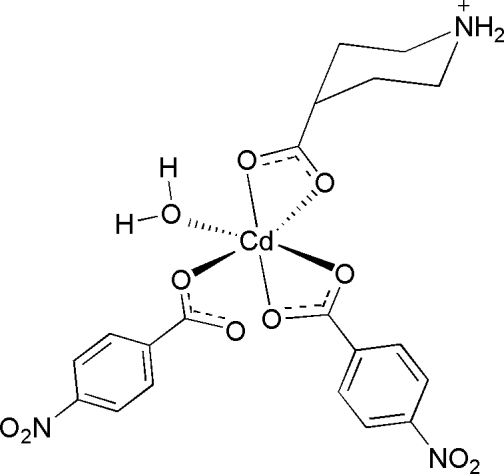


## Experimental

### 

#### Crystal data

[Cd(C_6_H_11_NO_2_)(C_7_H_4_NO_4_)_2_(H_2_O)]
*M* *_r_* = 591.80
Monoclinic, 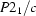

*a* = 22.7135 (7) Å
*b* = 6.6294 (2) Å
*c* = 14.9658 (5) Å
β = 91.3400 (10)°
*V* = 2252.89 (12) Å^3^
*Z* = 4
Mo *K*α radiation
μ = 1.04 mm^−1^
*T* = 296 K
0.32 × 0.28 × 0.22 mm

#### Data collection

Bruker APEXII CCD area-detector diffractometer
Absorption correction: multi-scan (*SADABS*; Sheldrick, 1996 ▶) *T*_min_ = 0.733, *T*_max_ = 0.804
10944 measured reflections
3950 independent reflections
3615 reflections with *I* > 2σ(*I*)
*R*_int_ = 0.014

#### Refinement

*R*[*F* ^2^ > 2σ(*F* ^2^)] = 0.020
*wR*(*F* ^2^) = 0.056
*S* = 1.05
3950 reflections
316 parameters
18 restraints
H-atom parameters constrained
Δρ_max_ = 0.37 e Å^−3^
Δρ_min_ = −0.32 e Å^−3^

### 

Data collection: *APEX2* (Bruker, 2003 ▶); cell refinement: *SAINT* (Bruker, 2001 ▶); data reduction: *SAINT*; program(s) used to solve structure: *SHELXS97* (Sheldrick, 2008 ▶); program(s) used to refine structure: *SHELXL97* (Sheldrick, 2008 ▶); molecular graphics: *SHELXTL* (Sheldrick, 2008 ▶) and *DIAMOND* (Brandenburg & Berndt, 1999 ▶); software used to prepare material for publication: *SHELXL97*.

## Supplementary Material

Crystal structure: contains datablocks I, global. DOI: 10.1107/S160053680901811X/bq2139sup1.cif

Structure factors: contains datablocks I. DOI: 10.1107/S160053680901811X/bq2139Isup2.hkl

Additional supplementary materials:  crystallographic information; 3D view; checkCIF report

## Figures and Tables

**Table 1:** Selected geometric parameters (Å, °)

Cd1—O5	2.1917 (18)
Cd1—O11	2.3061 (19)
Cd1—O2	2.3229 (17)
Cd1—O9	2.3327 (17)
Cd1—O10	2.3534 (18)
Cd1—O1	2.3684 (19)

**Table 2:** Hydrogen-bond geometry (Å, °)

*D*—H⋯*A*	*D*—H	H⋯*A*	*D*⋯*A*	*D*—H⋯*A*
O11—H11*A*⋯O3^i^	0.85	2.26	3.019 (3)	149
O11—H11*B*⋯O10^ii^	0.85	1.91	2.754 (3)	172
N3—H3*A*⋯O9^iii^	0.90	2.04	2.887 (3)	156
N3—H3*B*⋯O6^iv^	0.90	1.89	2.762 (3)	163

Symmetry codes: (i) 
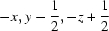
; (ii) 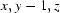; (iii) 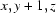; (iv) 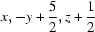.

**Fig. 1.**
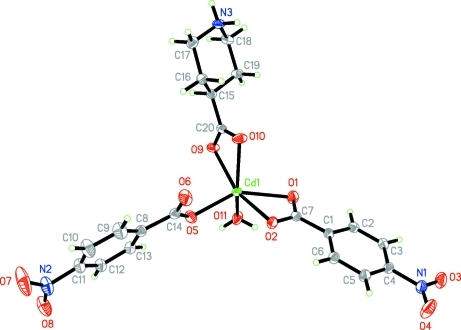
The molecular structure of (I). Displacement ellipsoids are drawn at the 30% probability level.

**Fig. 2.**
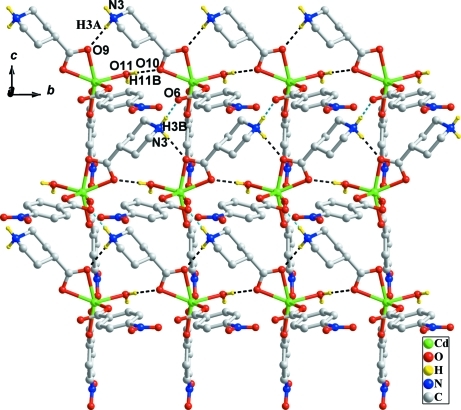
The 2-D layer of (I) formed by hydrogen bonding interactions. Only H atoms involved in hydrogen bonds are included.

**Fig. 3.**
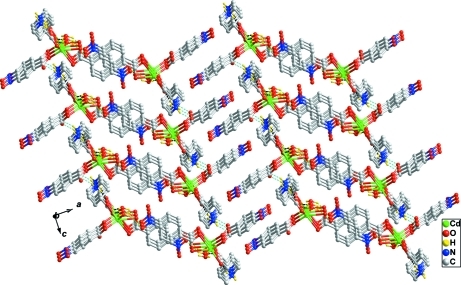
The 3-D network of (I) formed by hydrogen bond interactions. Only H atoms involved in hydrogen bonds are included.

## supplementary crystallographic information

### Comment

Recently, the rational design and skillful construction of the coordination complexes with mixed ligands have aroused great interest due to their intriguing framework topologies and potential applications in ino-exchange (Muthu *et al.*, 2002), catalysis (Fujita *et al.*, 1994), luminescence (Zheng *et al.*, 2004), and gas storage (Rosi *et al.,* 2003). Bearing two potential binding sites (–NH– and –COOH) capable of coordination with transition metal atoms, 4-piperidinecarboxylic acid exists as a zwitterion with the amino group protonated and the carboxylic group deprotonated in aqueous solution (Mora *et al.*, 2002). Thus, by carefully control the degree of the protonation/deprotonation of carboxylic and/or amino groups, it can selectively act as a bridge ligand linking different metal atoms into an infinite high-dimensional framework or as a terminal ligand forming a discrete complex (Inomata *et al.*, 2002). However, to the best of our knowledge, only a few examples involved in 4-piperidinecarboxylic acid have been reported by far (Adams *et al.*, 2006*a*,*b*; Podesta *et al.*, 2002). Thus, to continue to explore the coordination behavior of 4-piperidinecarboxylic acid, herein, we report the crystal structure of a Cd^II^ complex with 4-piperidinecarboxylate and 4-nitrobenzoxylate anion.

As shown in Fig. 1, the mononuclear structure of the title complex, (I), consists of one crystallographic independent Cd^II^ atom, two separate 4-nitrobenzoxylate anions, one zwitterionic 4-piperidinecarboxylate molecule, and one coordinated water molecule. The sole Cd^II ^center is six-coordinated by one coordinated water molecule (O11), and five carboxylate O atoms from two independent 4-nitrobenzoxylate (O1, O2 and O5) and one 4-piperidinecarboxylate molecule (O9 and O10), exhibiting a distorted octahedral geometry (Table 1.). The Cd–O bond distances are in the range of 2.1914 (16)–2.3684 (17)Å, which are comparable to those previously reported values (Inomata *et al.*, 2004; Wang *et al., *2008). The carboxylate group of 4-nitrobenzoxylate anion presents two different coordination modes: monodentate and asymmetric chelating bidentate fashions. In contrast, the carboxylate group of 4-piperidinecarboxylate only adopts an asymmetric chelating bidentate coordination mode. Additionally, the N atom of piperidine ring does not coordinate to a metal atom, and the protonated piperidine ring of 4-piperdinecarboxylate is in a chair conformation significantly resulted from the relatively lower energy (Delgado *et al.*, 2001).

As shown in Fig. 2, the adjacent mononuclear entities are linked in to a two-dimensional (2-D) layer by threefold hydrogen bonding interactions between the protonated –NH_2_^+^/coordinated water molecule and the carboxylate group of 4-piperdinecarboxylate /4-nitrobenzoxylate anion (Table 2.). The neighbour 2-D layers are further assembled into a 3-D supramolecular network (Fig. 3) also by the hydrogen bonding interaction between the coordinated water molecule and the carboxylate group of 4-nitrobenzoxylate anion (O11–H11A···O3). Thus, the abundant hydrogen bonds interactions significantly dominate the formation of 3-D supramolecular network of the title complex.

### Experimental

To an aqueous solution (5 ml) of CdCl_2_. 2.5H_2_O (45.7 mg, 0.2 mmol) was slowly added a methanol solution (5 ml) containing 4-piperidinecarboxylic acid (12.9 mg, 0.1 mmol) and 4-nitrobenzoic acid (16.7 mg, 0.1 mmol) with constant stirring. And the pH value of the mixture was adjusted to 6 by NaOH solution (0.1 *M*). The resulting colorless solution was further stirred for half an hour and filtered. The filtrate was allowed to evaporate at room temperature. Pale-yellow block-shaped crystals were obtained within two weeks (yield 70% based on Cd^II^ salt).

### Refinement

The H atoms of the water molecule were located in the final difference Fourier map, their positions were refined and their isotropic displacement parameters were set to 1.5 times the equivalent displacement parameter of the O atom. H atoms of the NH_2_^+ ^and the piperidine ring were placed in geometrically calculated positions and their isotropic displacement parameters were set to 1.2 times the equivalent displacement parameter of their parent atoms.

### Crystal data

**Table:**

[Cd(C_6_H_11_NO_2_)(C_7_H_4_NO_4_)_2_(H_2_O)]	*F*(000) = 1192
*M**_r_* = 591.80	*D*_x_ = 1.745 Mg m^−^^3^
Monoclinic, *P*2_1_/*c*	Mo *K*α radiation, λ = 0.71073 Å
Hall symbol: -P 2ybc	Cell parameters from 8249 reflections
*a* = 22.7135 (7) Å	θ = 2.9–27.8°
*b* = 6.6294 (2) Å	µ = 1.04 mm^−^^1^
*c* = 14.9658 (5) Å	*T* = 296 K
β = 91.340 (1)°	Block, pale-yellow
*V* = 2252.89 (12) Å^3^	0.32 × 0.28 × 0.22 mm
*Z* = 4	

### Data collection

**Table:**

Bruker APEXII CCD area-detector diffractometer	3950 independent reflections
Radiation source: fine-focus sealed tube	3615 reflections with *I* > 2σ(*I*)
graphite	*R*_int_ = 0.014
φ and ω scans	θ_max_ = 25.0°, θ_min_ = 0.9°
Absorption correction: multi-scan (*SADABS*; Sheldrick, 1996)	*h* = −18→26
*T*_min_ = 0.733, *T*_max_ = 0.804	*k* = −7→7
10944 measured reflections	*l* = −17→17

### Refinement

**Table:**

Refinement on *F*^2^	Primary atom site location: structure-invariant direct methods
Least-squares matrix: full	Secondary atom site location: difference Fourier map
*R*[*F*^2^ > 2σ(*F*^2^)] = 0.020	Hydrogen site location: inferred from neighbouring sites
*wR*(*F*^2^) = 0.056	H-atom parameters constrained
*S* = 1.05	*w* = 1/[σ^2^(*F*_o_^2^) + (0.0248*P*)^2^ + 1.8541*P*] where *P* = (*F*_o_^2^ + 2*F*_c_^2^)/3
3950 reflections	(Δ/σ)_max_ < 0.001
316 parameters	Δρ_max_ = 0.37 e Å^−^^3^
18 restraints	Δρ_min_ = −0.32 e Å^−^^3^

### Special details

**Table:**

Geometry. All e.s.d.'s (except the e.s.d. in the dihedral angle between two l.s. planes) are estimated using the full covariance matrix. The cell e.s.d.'s are taken into account individually in the estimation of e.s.d.'s in distances, angles and torsion angles; correlations between e.s.d.'s in cell parameters are only used when they are defined by crystal symmetry. An approximate (isotropic) treatment of cell e.s.d.'s is used for estimating e.s.d.'s involving l.s. planes.
Refinement. Refinement of *F*^2^ against ALL reflections. The weighted *R*-factor *wR* and goodness of fit *S* are based on *F*^2^, conventional *R*-factors *R* are based on *F*, with *F* set to zero for negative *F*^2^. The threshold expression of *F*^2^ > σ(*F*^2^) is used only for calculating *R*-factors(gt) *etc*. and is not relevant to the choice of reflections for refinement. *R*-factors based on *F*^2^ are statistically about twice as large as those based on *F*, and *R*- factors based on ALL data will be even larger.

### Fractional atomic coordinates and isotropic or equivalent isotropic displacement parameters (Å^2^)

**Table:**

	*x*	*y*	*z*	*U*_iso_*/*U*_eq_	
Cd1	0.219460 (8)	0.68670 (3)	0.490995 (11)	0.03129 (7)	
O1	0.11917 (8)	0.7419 (3)	0.45149 (12)	0.0458 (5)	
O2	0.18499 (8)	0.7480 (3)	0.34618 (12)	0.0372 (4)	
O3	−0.10379 (9)	0.7684 (4)	0.13243 (15)	0.0595 (6)	
O4	−0.04142 (11)	0.7829 (5)	0.02901 (15)	0.0822 (9)	
O5	0.30087 (8)	0.5260 (3)	0.46044 (13)	0.0450 (5)	
O6	0.34464 (10)	0.7998 (3)	0.40672 (16)	0.0563 (6)	
O7	0.59387 (12)	0.2162 (5)	0.3509 (3)	0.1201 (13)	
O8	0.54181 (11)	−0.0455 (4)	0.3661 (2)	0.0759 (7)	
O9	0.24780 (8)	0.7579 (3)	0.63847 (11)	0.0371 (4)	
O10	0.23667 (10)	1.0130 (3)	0.54773 (12)	0.0506 (5)	
O11	0.18002 (9)	0.3782 (3)	0.52820 (14)	0.0490 (5)	
H11A	0.1486	0.3692	0.4963	0.073*	
H11B	0.2007	0.2720	0.5342	0.073*	
N1	−0.05315 (11)	0.7738 (4)	0.10707 (16)	0.0432 (6)	
N2	0.54786 (12)	0.1355 (4)	0.3662 (2)	0.0637 (8)	
N3	0.28125 (9)	1.4822 (3)	0.78005 (14)	0.0354 (5)	
H3A	0.2604	1.5610	0.7420	0.043*	
H3B	0.2946	1.5603	0.8253	0.043*	
C1	0.08383 (11)	0.7676 (4)	0.30125 (16)	0.0297 (5)	
C2	0.02551 (11)	0.7688 (4)	0.32711 (17)	0.0326 (5)	
H2	0.0169	0.7675	0.3875	0.039*	
C3	−0.01979 (11)	0.7717 (4)	0.26380 (17)	0.0342 (6)	
H3	−0.0589	0.7730	0.2806	0.041*	
C4	−0.00508 (11)	0.7727 (4)	0.17489 (17)	0.0331 (6)	
C5	0.05237 (12)	0.7745 (4)	0.14668 (17)	0.0384 (6)	
H5	0.0608	0.7779	0.0862	0.046*	
C6	0.09691 (11)	0.7712 (4)	0.21095 (17)	0.0354 (6)	
H6	0.1360	0.7713	0.1937	0.043*	
C7	0.13244 (11)	0.7537 (4)	0.37115 (17)	0.0309 (5)	
C8	0.39914 (11)	0.4942 (4)	0.41654 (17)	0.0356 (6)	
C9	0.44834 (14)	0.5789 (5)	0.3783 (2)	0.0549 (8)	
H9	0.4480	0.7144	0.3623	0.066*	
C10	0.49774 (14)	0.4642 (5)	0.3638 (3)	0.0622 (9)	
H10	0.5312	0.5217	0.3398	0.075*	
C11	0.49655 (12)	0.2628 (5)	0.3857 (2)	0.0445 (7)	
C12	0.44856 (12)	0.1744 (4)	0.42363 (18)	0.0402 (6)	
H12	0.4488	0.0377	0.4377	0.048*	
C13	0.39998 (12)	0.2921 (4)	0.44039 (17)	0.0367 (6)	
H13	0.3676	0.2356	0.4679	0.044*	
C14	0.34426 (12)	0.6197 (4)	0.42904 (17)	0.0372 (6)	
C15	0.27278 (11)	1.0862 (4)	0.69599 (15)	0.0306 (5)	
H15	0.2954	1.0078	0.7404	0.037*	
C16	0.31251 (11)	1.2505 (4)	0.66016 (17)	0.0349 (6)	
H16A	0.3469	1.1889	0.6342	0.042*	
H16B	0.2915	1.3242	0.6133	0.042*	
C17	0.33216 (12)	1.3960 (4)	0.73289 (18)	0.0399 (6)	
H17A	0.3577	1.3262	0.7756	0.048*	
H17B	0.3546	1.5045	0.7067	0.048*	
C18	0.24177 (13)	1.3237 (4)	0.81621 (17)	0.0370 (6)	
H18A	0.2081	1.3870	0.8434	0.044*	
H18B	0.2629	1.2472	0.8620	0.044*	
C19	0.22071 (11)	1.1830 (4)	0.74276 (17)	0.0341 (6)	
H19A	0.1969	1.2576	0.6994	0.041*	
H19B	0.1963	1.0783	0.7679	0.041*	
C20	0.25127 (11)	0.9437 (4)	0.62321 (16)	0.0326 (5)	

### Atomic displacement parameters (Å^2^)

**Table:**

	*U*^11^	*U*^22^	*U*^33^	*U*^12^	*U*^13^	*U*^23^
Cd1	0.03884 (12)	0.02632 (11)	0.02842 (11)	0.00414 (8)	−0.00520 (7)	−0.00244 (7)
O1	0.0443 (11)	0.0636 (13)	0.0294 (10)	0.0109 (10)	−0.0012 (8)	0.0033 (9)
O2	0.0321 (10)	0.0429 (10)	0.0364 (10)	0.0004 (8)	−0.0041 (8)	0.0006 (8)
O3	0.0341 (12)	0.0792 (16)	0.0646 (14)	0.0025 (11)	−0.0104 (10)	0.0013 (12)
O4	0.0595 (15)	0.149 (3)	0.0372 (13)	0.0001 (16)	−0.0105 (11)	0.0044 (15)
O5	0.0403 (11)	0.0396 (11)	0.0552 (12)	0.0066 (9)	0.0049 (9)	−0.0040 (9)
O6	0.0592 (13)	0.0369 (12)	0.0725 (15)	0.0100 (10)	−0.0061 (11)	0.0148 (10)
O7	0.0492 (15)	0.0736 (19)	0.240 (4)	0.0041 (14)	0.053 (2)	0.026 (2)
O8	0.0572 (14)	0.0516 (15)	0.120 (2)	0.0142 (12)	0.0266 (14)	0.0137 (14)
O9	0.0543 (11)	0.0239 (9)	0.0331 (9)	−0.0009 (8)	−0.0006 (8)	−0.0017 (7)
O10	0.0843 (14)	0.0275 (9)	0.0388 (10)	0.0071 (9)	−0.0225 (10)	−0.0030 (8)
O11	0.0479 (11)	0.0328 (10)	0.0650 (13)	−0.0032 (9)	−0.0228 (10)	0.0083 (9)
N1	0.0420 (14)	0.0409 (13)	0.0462 (15)	0.0022 (11)	−0.0101 (11)	0.0018 (11)
N2	0.0408 (14)	0.0533 (16)	0.098 (2)	0.0069 (12)	0.0211 (14)	0.0190 (15)
N3	0.0487 (13)	0.0225 (10)	0.0348 (11)	−0.0030 (9)	−0.0047 (9)	−0.0030 (9)
C1	0.0331 (13)	0.0233 (12)	0.0325 (13)	0.0020 (10)	−0.0009 (10)	0.0021 (10)
C2	0.0384 (14)	0.0297 (13)	0.0299 (13)	0.0030 (11)	0.0027 (11)	0.0025 (10)
C3	0.0301 (13)	0.0307 (13)	0.0418 (14)	0.0015 (10)	0.0034 (11)	0.0024 (11)
C4	0.0350 (14)	0.0280 (13)	0.0359 (14)	0.0012 (10)	−0.0067 (11)	0.0015 (10)
C5	0.0400 (15)	0.0461 (16)	0.0290 (13)	0.0027 (12)	0.0021 (11)	0.0029 (12)
C6	0.0306 (13)	0.0420 (15)	0.0338 (13)	0.0004 (11)	0.0028 (11)	0.0013 (11)
C7	0.0365 (14)	0.0211 (12)	0.0348 (14)	0.0027 (10)	−0.0035 (11)	0.0000 (10)
C8	0.0371 (14)	0.0330 (14)	0.0364 (13)	0.0006 (11)	−0.0047 (11)	0.0007 (11)
C9	0.0538 (19)	0.0326 (16)	0.079 (2)	−0.0024 (14)	0.0086 (16)	0.0141 (15)
C10	0.0452 (18)	0.0460 (19)	0.096 (3)	−0.0031 (15)	0.0208 (17)	0.0118 (18)
C11	0.0355 (15)	0.0420 (16)	0.0563 (18)	0.0032 (12)	0.0044 (13)	0.0059 (14)
C12	0.0409 (15)	0.0327 (14)	0.0469 (16)	0.0016 (12)	0.0012 (12)	0.0075 (12)
C13	0.0357 (14)	0.0367 (15)	0.0376 (14)	−0.0008 (11)	0.0015 (11)	0.0052 (11)
C14	0.0428 (15)	0.0368 (15)	0.0316 (13)	0.0036 (12)	−0.0089 (11)	−0.0015 (11)
C15	0.0397 (14)	0.0226 (12)	0.0293 (12)	0.0040 (10)	−0.0045 (10)	0.0004 (10)
C16	0.0326 (13)	0.0373 (14)	0.0348 (13)	0.0010 (11)	0.0039 (11)	−0.0015 (11)
C17	0.0365 (14)	0.0382 (15)	0.0447 (15)	−0.0071 (12)	−0.0039 (12)	0.0011 (12)
C18	0.0517 (16)	0.0265 (13)	0.0332 (13)	−0.0015 (11)	0.0082 (12)	−0.0019 (10)
C19	0.0386 (14)	0.0273 (13)	0.0367 (14)	−0.0066 (11)	0.0064 (11)	−0.0020 (11)
C20	0.0410 (13)	0.0248 (12)	0.0318 (12)	0.0055 (10)	−0.0035 (10)	−0.0039 (10)

### Geometric parameters (Å, °)

**Table:**

Cd1—O5	2.1917 (18)	C3—C4	1.379 (4)
Cd1—O11	2.3061 (19)	C3—H3	0.9300
Cd1—O2	2.3229 (17)	C4—C5	1.381 (4)
Cd1—O9	2.3327 (17)	C5—C6	1.380 (4)
Cd1—O10	2.3534 (18)	C5—H5	0.9300
Cd1—O1	2.3684 (19)	C6—H6	0.9300
Cd1—C7	2.675 (2)	C8—C9	1.386 (4)
Cd1—C20	2.697 (2)	C8—C13	1.387 (4)
O1—C7	1.249 (3)	C8—C14	1.514 (4)
O2—C7	1.260 (3)	C9—C10	1.377 (4)
O3—N1	1.220 (3)	C9—H9	0.9300
O4—N1	1.206 (3)	C10—C11	1.375 (4)
O5—C14	1.265 (3)	C10—H10	0.9300
O6—C14	1.240 (3)	C11—C12	1.372 (4)
O7—N2	1.201 (4)	C12—C13	1.379 (4)
O8—N2	1.207 (4)	C12—H12	0.9300
O9—C20	1.256 (3)	C13—H13	0.9300
O10—C20	1.257 (3)	C15—C20	1.514 (3)
O11—H11A	0.8504	C15—C16	1.520 (4)
O11—H11B	0.8500	C15—C19	1.529 (3)
N1—C4	1.473 (3)	C15—H15	0.9800
N2—C11	1.474 (4)	C16—C17	1.514 (4)
N3—C17	1.483 (3)	C16—H16A	0.9700
N3—C18	1.491 (3)	C16—H16B	0.9700
N3—H3A	0.9000	C17—H17A	0.9700
N3—H3B	0.9000	C17—H17B	0.9700
C1—C2	1.389 (4)	C18—C19	1.511 (3)
C1—C6	1.391 (3)	C18—H18A	0.9700
C1—C7	1.506 (3)	C18—H18B	0.9700
C2—C3	1.382 (4)	C19—H19A	0.9700
C2—H2	0.9300	C19—H19B	0.9700
			
O5—Cd1—O11	87.34 (7)	C1—C6—H6	119.7
O5—Cd1—O2	99.09 (7)	O1—C7—O2	122.4 (2)
O11—Cd1—O2	104.84 (7)	O1—C7—C1	118.9 (2)
O5—Cd1—O9	94.60 (7)	O2—C7—C1	118.7 (2)
O11—Cd1—O9	92.93 (7)	O1—C7—Cd1	62.30 (13)
O2—Cd1—O9	157.95 (7)	O2—C7—Cd1	60.23 (12)
O5—Cd1—O10	112.86 (8)	C1—C7—Cd1	173.83 (17)
O11—Cd1—O10	142.21 (7)	C9—C8—C13	119.4 (3)
O2—Cd1—O10	103.02 (6)	C9—C8—C14	120.2 (2)
O9—Cd1—O10	55.45 (6)	C13—C8—C14	120.4 (2)
O5—Cd1—O1	146.54 (7)	C10—C9—C8	120.6 (3)
O11—Cd1—O1	79.66 (7)	C10—C9—H9	119.7
O2—Cd1—O1	55.86 (6)	C8—C9—H9	119.7
O9—Cd1—O1	116.58 (6)	C11—C10—C9	118.6 (3)
O10—Cd1—O1	95.67 (7)	C11—C10—H10	120.7
O5—Cd1—C7	123.68 (7)	C9—C10—H10	120.7
O11—Cd1—C7	91.37 (7)	C12—C11—C10	122.3 (3)
O2—Cd1—C7	28.08 (7)	C12—C11—N2	118.4 (3)
O9—Cd1—C7	141.64 (7)	C10—C11—N2	119.3 (3)
O10—Cd1—C7	101.72 (7)	C11—C12—C13	118.7 (3)
O1—Cd1—C7	27.83 (7)	C11—C12—H12	120.7
O5—Cd1—C20	104.32 (7)	C13—C12—H12	120.7
O11—Cd1—C20	118.82 (7)	C12—C13—C8	120.4 (3)
O2—Cd1—C20	130.75 (7)	C12—C13—H13	119.8
O9—Cd1—C20	27.72 (7)	C8—C13—H13	119.8
O10—Cd1—C20	27.77 (7)	O6—C14—O5	125.7 (3)
O1—Cd1—C20	108.98 (7)	O6—C14—C8	119.0 (3)
C7—Cd1—C20	124.63 (7)	O5—C14—C8	115.3 (2)
C7—O1—Cd1	89.86 (15)	C20—C15—C16	112.2 (2)
C7—O2—Cd1	91.69 (15)	C20—C15—C19	110.6 (2)
C14—O5—Cd1	120.47 (17)	C16—C15—C19	109.4 (2)
C20—O9—Cd1	92.50 (14)	C20—C15—H15	108.2
C20—O10—Cd1	91.50 (15)	C16—C15—H15	108.2
Cd1—O11—H11A	104.6	C19—C15—H15	108.2
Cd1—O11—H11B	122.9	C17—C16—C15	111.8 (2)
H11A—O11—H11B	117.1	C17—C16—H16A	109.3
O4—N1—O3	122.3 (2)	C15—C16—H16A	109.3
O4—N1—C4	119.4 (2)	C17—C16—H16B	109.3
O3—N1—C4	118.3 (2)	C15—C16—H16B	109.3
O7—N2—O8	122.8 (3)	H16A—C16—H16B	107.9
O7—N2—C11	118.6 (3)	N3—C17—C16	111.6 (2)
O8—N2—C11	118.6 (3)	N3—C17—H17A	109.3
C17—N3—C18	112.6 (2)	C16—C17—H17A	109.3
C17—N3—H3A	109.1	N3—C17—H17B	109.3
C18—N3—H3A	109.1	C16—C17—H17B	109.3
C17—N3—H3B	109.1	H17A—C17—H17B	108.0
C18—N3—H3B	109.1	N3—C18—C19	110.7 (2)
H3A—N3—H3B	107.8	N3—C18—H18A	109.5
C2—C1—C6	119.8 (2)	C19—C18—H18A	109.5
C2—C1—C7	119.7 (2)	N3—C18—H18B	109.5
C6—C1—C7	120.4 (2)	C19—C18—H18B	109.5
C3—C2—C1	120.6 (2)	H18A—C18—H18B	108.1
C3—C2—H2	119.7	C18—C19—C15	110.9 (2)
C1—C2—H2	119.7	C18—C19—H19A	109.5
C4—C3—C2	117.9 (2)	C15—C19—H19A	109.5
C4—C3—H3	121.0	C18—C19—H19B	109.5
C2—C3—H3	121.0	C15—C19—H19B	109.5
C3—C4—C5	123.1 (2)	H19A—C19—H19B	108.0
C3—C4—N1	118.2 (2)	O9—C20—O10	120.4 (2)
C5—C4—N1	118.7 (2)	O9—C20—C15	120.1 (2)
C6—C5—C4	118.0 (2)	O10—C20—C15	119.5 (2)
C6—C5—H5	121.0	O9—C20—Cd1	59.78 (12)
C4—C5—H5	121.0	O10—C20—Cd1	60.73 (13)
C5—C6—C1	120.5 (2)	C15—C20—Cd1	176.71 (18)
C5—C6—H6	119.7		
			
O5—Cd1—O1—C7	−44.7 (2)	C20—Cd1—C7—O2	113.00 (15)
O11—Cd1—O1—C7	−113.45 (16)	O5—Cd1—C7—C1	49.5 (17)
O2—Cd1—O1—C7	2.54 (14)	O11—Cd1—C7—C1	−38.2 (17)
O9—Cd1—O1—C7	158.45 (14)	O2—Cd1—C7—C1	81.7 (17)
O10—Cd1—O1—C7	104.44 (16)	O9—Cd1—C7—C1	−134.7 (17)
C20—Cd1—O1—C7	129.46 (15)	O10—Cd1—C7—C1	177.4 (17)
O5—Cd1—O2—C7	153.29 (15)	O1—Cd1—C7—C1	−102.8 (17)
O11—Cd1—O2—C7	63.65 (15)	C20—Cd1—C7—C1	−165.3 (17)
O9—Cd1—O2—C7	−79.0 (2)	C13—C8—C9—C10	0.1 (5)
O10—Cd1—O2—C7	−90.55 (15)	C14—C8—C9—C10	−177.8 (3)
O1—Cd1—O2—C7	−2.52 (14)	C8—C9—C10—C11	1.8 (5)
C20—Cd1—O2—C7	−88.77 (16)	C9—C10—C11—C12	−1.9 (5)
O11—Cd1—O5—C14	176.52 (19)	C9—C10—C11—N2	176.8 (3)
O2—Cd1—O5—C14	71.91 (19)	O7—N2—C11—C12	−164.7 (4)
O9—Cd1—O5—C14	−90.75 (19)	O8—N2—C11—C12	15.5 (5)
O10—Cd1—O5—C14	−36.4 (2)	O7—N2—C11—C10	16.6 (5)
O1—Cd1—O5—C14	109.9 (2)	O8—N2—C11—C10	−163.1 (4)
C7—Cd1—O5—C14	86.6 (2)	C10—C11—C12—C13	−0.1 (5)
C20—Cd1—O5—C14	−64.4 (2)	N2—C11—C12—C13	−178.8 (3)
O5—Cd1—O9—C20	112.35 (16)	C11—C12—C13—C8	2.1 (4)
O11—Cd1—O9—C20	−160.08 (16)	C9—C8—C13—C12	−2.1 (4)
O2—Cd1—O9—C20	−16.0 (3)	C14—C8—C13—C12	175.7 (2)
O10—Cd1—O9—C20	−2.34 (15)	Cd1—O5—C14—O6	−5.4 (4)
O1—Cd1—O9—C20	−80.19 (16)	Cd1—O5—C14—C8	176.17 (15)
C7—Cd1—O9—C20	−64.15 (19)	C9—C8—C14—O6	−1.8 (4)
O5—Cd1—O10—C20	−77.03 (17)	C13—C8—C14—O6	−179.6 (3)
O11—Cd1—O10—C20	40.4 (2)	C9—C8—C14—O5	176.7 (3)
O2—Cd1—O10—C20	177.09 (16)	C13—C8—C14—O5	−1.1 (4)
O9—Cd1—O10—C20	2.33 (15)	C20—C15—C16—C17	178.2 (2)
O1—Cd1—O10—C20	120.86 (16)	C19—C15—C16—C17	55.1 (3)
C7—Cd1—O10—C20	148.36 (16)	C18—N3—C17—C16	54.5 (3)
C6—C1—C2—C3	0.6 (4)	C15—C16—C17—N3	−54.3 (3)
C7—C1—C2—C3	−176.9 (2)	C17—N3—C18—C19	−56.0 (3)
C1—C2—C3—C4	0.2 (4)	N3—C18—C19—C15	57.1 (3)
C2—C3—C4—C5	−1.2 (4)	C20—C15—C19—C18	179.4 (2)
C2—C3—C4—N1	179.4 (2)	C16—C15—C19—C18	−56.6 (3)
O4—N1—C4—C3	177.0 (3)	Cd1—O9—C20—O10	4.2 (3)
O3—N1—C4—C3	−1.9 (4)	Cd1—O9—C20—C15	−176.2 (2)
O4—N1—C4—C5	−2.4 (4)	Cd1—O10—C20—O9	−4.1 (3)
O3—N1—C4—C5	178.7 (3)	Cd1—O10—C20—C15	176.2 (2)
C3—C4—C5—C6	1.3 (4)	C16—C15—C20—O9	140.5 (2)
N1—C4—C5—C6	−179.3 (2)	C19—C15—C20—O9	−97.1 (3)
C4—C5—C6—C1	−0.5 (4)	C16—C15—C20—O10	−39.9 (3)
C2—C1—C6—C5	−0.4 (4)	C19—C15—C20—O10	82.6 (3)
C7—C1—C6—C5	177.0 (2)	C16—C15—C20—Cd1	53 (3)
Cd1—O1—C7—O2	−4.6 (3)	C19—C15—C20—Cd1	175 (3)
Cd1—O1—C7—C1	173.1 (2)	O5—Cd1—C20—O9	−72.07 (16)
Cd1—O2—C7—O1	4.7 (3)	O11—Cd1—C20—O9	22.85 (18)
Cd1—O2—C7—C1	−173.03 (19)	O2—Cd1—C20—O9	172.13 (13)
C2—C1—C7—O1	0.5 (4)	O10—Cd1—C20—O9	175.9 (3)
C6—C1—C7—O1	−176.9 (2)	O1—Cd1—C20—O9	111.27 (15)
C2—C1—C7—O2	178.3 (2)	C7—Cd1—C20—O9	137.25 (15)
C6—C1—C7—O2	0.9 (4)	O5—Cd1—C20—O10	112.06 (16)
C2—C1—C7—Cd1	100.0 (17)	O11—Cd1—C20—O10	−153.02 (15)
C6—C1—C7—Cd1	−77.4 (17)	O2—Cd1—C20—O10	−3.7 (2)
O5—Cd1—C7—O1	152.24 (15)	O9—Cd1—C20—O10	−175.9 (3)
O11—Cd1—C7—O1	64.52 (16)	O1—Cd1—C20—O10	−64.60 (17)
O2—Cd1—C7—O1	−175.5 (2)	C7—Cd1—C20—O10	−38.62 (19)
O9—Cd1—C7—O1	−31.9 (2)	O5—Cd1—C20—C15	17 (3)
O10—Cd1—C7—O1	−79.80 (16)	O11—Cd1—C20—C15	112 (3)
C20—Cd1—C7—O1	−62.53 (18)	O2—Cd1—C20—C15	−99 (3)
O5—Cd1—C7—O2	−32.23 (17)	O9—Cd1—C20—C15	89 (3)
O11—Cd1—C7—O2	−119.95 (15)	O10—Cd1—C20—C15	−95 (3)
O9—Cd1—C7—O2	143.58 (14)	O1—Cd1—C20—C15	−159 (3)
O10—Cd1—C7—O2	95.73 (15)	C7—Cd1—C20—C15	−133 (3)
O1—Cd1—C7—O2	175.5 (2)		

### Hydrogen-bond geometry (Å, °)

**Table:**

*D*—H···*A*	*D*—H	H···*A*	*D*···*A*	*D*—H···*A*
O11—H11A···O3^i^	0.85	2.26	3.019 (3)	149
O11—H11B···O10^ii^	0.85	1.91	2.754 (3)	172
N3—H3A···O9^iii^	0.90	2.04	2.887 (3)	156
N3—H3B···O6^iv^	0.90	1.89	2.762 (3)	163

Symmetry codes: (i) −*x*, *y*−1/2, −*z*+1/2; (ii) *x*, *y*−1, *z*; (iii) *x*, *y*+1, *z*; (iv) *x*, −*y*+5/2, *z*+1/2.
